# Accelerating adverse pregnancy outcomes research amidst rising medication use: parallel retrospective cohort analyses for signal prioritization

**DOI:** 10.1186/s12916-024-03717-0

**Published:** 2024-10-25

**Authors:** Yeon Mi Hwang, Samantha N. Piekos, Alison G. Paquette, Qi Wei, Nathan D. Price, Leroy Hood, Jennifer J. Hadlock

**Affiliations:** 1 Institute for Systems Biology, Seattle, WA USA; 2 Molecular Engineering & Sciences Institute, University of Washington, Seattle, WA USA; 3 Center for Biomedical Informatics Research, Stanford University School of Medicine, Stanford, CA USA; 4 Center for Developmental Biology and Regenerative Medicine, Seattle Children’s Research Institute, Seattle, WA USA; 5 Department of Pediatrics, Division of Genetic Medicine, School of Medicine, University of Washington, Seattle, WA USA; 6 Buck Institute for Research On Aging, Novato, CA USA; 7 Thorne Healthtech, New York, NY USA; 8 Phenome Health, Seattle, WA USA; 9 Department of Biomedical Informatics and Medical Education, School of Medicine, University of Washington, Seattle, WA USA

**Keywords:** Drug-related side effects and adverse reactions, Pharmacoepidemiology, Data mining, Pregnancy, Preterm birth

## Abstract

**Background:**

Pregnant women are significantly underrepresented in clinical trials, yet most of them take medication during pregnancy despite the limited safety data. The objective of this study was to characterize medication use during pregnancy and apply propensity score matching method at scale on patient records to accelerate and prioritize the drug effect signal detection associated with the risk of preterm birth and other adverse pregnancy outcomes.

**Methods:**

This was a retrospective study on continuously enrolled women who delivered live births between 2013/01/01 and 2022/12/31 (*n* = 365,075) at Providence St. Joseph Health. Our exposures of interest were all outpatient medications prescribed during pregnancy. We limited our analyses to medication that met the minimal sample size (*n* = 600). The primary outcome of interest was preterm birth. Secondary outcomes of interest were small for gestational age and low birth weight. We used propensity score matching at scale to evaluate the risk of these adverse pregnancy outcomes associated with drug exposure after adjusting for demographics, pregnancy characteristics, and comorbidities.

**Results:**

The total medication prescription rate increased from 58.5 to 75.3% (*P* < 0.0001) from 2013 to 2022. The prevalence rate of preterm birth was 7.7%. One hundred seventy-five out of 1329 prenatally prescribed outpatient medications met the minimum sample size. We identified 58 medications statistically significantly associated with the risk of preterm birth (*P* ≤ 0.1; decreased: 12, increased: 46).

**Conclusions:**

Most pregnant women are prescribed medication during pregnancy. This highlights the need to utilize existing real-world data to enhance our knowledge of the safety of medications in pregnancy. We narrowed down from 1329 to 58 medications that showed statistically significant association with the risk of preterm birth even after addressing numerous covariates through propensity score matching. This data-driven approach demonstrated that multiple testable hypotheses in pregnancy pharmacology can be prioritized at scale and lays the foundation for application in other pregnancy outcomes.

**Supplementary Information:**

The online version contains supplementary material available at 10.1186/s12916-024-03717-0.

## Background

Pharmaceutical companies primarily rely on pre-marketing randomized clinical trials to prevent and assess adverse drug reactions (ADRs). Despite the effort, studies conducted on inpatient populations estimated a serious ADR incidence rate of 6.7% (*N* ≥ 2,216,000) with a fatality rate of 0.32% (*N* ≥ 106,000), placing ADRs as the fourth leading cause of morbidity and mortality in the United States (US) health care systems [1, 2]. The incidence rate of ADRs in outpatients is harder to estimate, with studies suggesting rates ranging from 3 to 38% [3–8]. Estimated incidence rate of ADRs in both inpatient and outpatient demonstrates that unintended drug response is common and expected.

Pre-marketing random clinical trials rarely include pregnant women unless the product targets pregnant women [9]. Consequently, drug efficacy, safety, and dosages are determined based on data from men and non-pregnant women. While pregnant women are the most underrepresented population in clinical trials, they can experience some of the most complex medical situations. During pregnancy, women undergo marked physiological changes that significantly alter the pharmacokinetics and pharmacodynamics of drugs [10]. Therefore, current knowledge in pharmacology should not be directly applied to pregnant women, as inadequate information on the pharmacology of pregnancy exposes them to a high likelihood of experiencing unintended drug responses.

Despite the limited availability of safety information regarding medication use during pregnancy, many pregnant women continue to use medications. Overall, 93.9% of pregnant women take at least one medication (over-the-counter or prescribed) and typically use an average of 4.2 during pregnancy. Usage of prescribed medication by pregnant women varies globally, ranging from 23 to 96%, with the US in 2008 reporting a usage rate of 49% among pregnant women [11]. Given the prevalent use of medication among pregnant women and the challenges associated with conducting prospective clinical trials on this population, leveraging real-world data has emerged as a promising supplemental approach to investigate the effects of drugs during pregnancy. Electronic health records (EHRs) are particularly suitable candidates among these real-world data sources. EHRs contain rich and comprehensive information about patients' longitudinal health profiles, potential confounding factors, and prescription history. Active research on developing novel methodologies for not only ADRs [12, 13] but also for drug repositioning [14] and drug-drug interactions [15, 16] is ongoing.

However, despite these advancements in data-driven healthcare research, the field of pregnancy research has been slower in adopting these novel methodologies. In summary, there is a pressing need to establish a foundational framework for systematically investigating drug responses during pregnancy at scale using real-world data. Such an effort is crucial, as it can lead to the generation of testable hypotheses related to drug effects on pregnancy outcomes, both positive and negative. Furthermore, uncovering drug responses that do not pose risks to adverse pregnancy outcomes can provide valuable insights into drug safety during pregnancy. Here, we selected preterm birth (PTB) as our primary outcome of interest. PTB, defined as birth occurring before 37 weeks of gestation, significantly contributes to perinatal morbidity and mortality in developed countries. PTB accounts for 75% of perinatal mortality cases and over half of long-term morbidity [17].

We employed a large-scale propensity score matching approach on patient records to expedite the generation and prioritization of testable hypotheses related to the risk of PTB. We hypothesized there exist not yet characterized pharmacological signals with medication and risk of PTB. Beyond hypothesis generation, we investigated a few detected drug effect signals using traditional pharmacoepidemiology methods.

## Methods

### Study design, setting, and participants

Providence St. Joseph Health (PSJH) is an integrated US community healthcare system that provides care in urban and rural settings across seven states: Alaska, California, Montana, Oregon, New Mexico, Texas, and Washington. We used PSJH pregnant patient records who delivered live infants from January 1, 2013, through December 31, 2022 (*n* = 543,408). We excluded multiple pregnancies and deliveries with gestational age (GA) of less than 20 weeks (*n* = 516,881). GA was limited to 20 weeks or greater because ascertainment bias is particularly high for EHR data earlier in pregnancy. This study population may be biased toward lower-risk pregnancy cases. This is because high-risk pregnancy cases are often transferred to third-level academic medical centers. We excluded patients who were not continuously enrolled from 180 days before the start of pregnancy (last menstrual period, LMP) to the time of delivery (*n* = 365,075). Our definition of continuous enrollment was at least one encounter 180 days before LMP and one encounter on or after the delivery date. This was done to partially address surveillance bias.

All procedures were reviewed and approved by the Institutional Review Board at the PSJH through expedited review on 11–04-2020 (study number STUDY2020000196). Consent was waived because disclosure of protected health information for the study involved no more than minimal risk to the privacy of individuals.

### Variables

#### Exposures

We mapped all prescription records during pregnancy to the RxNorm code based on ingredients. We split the cohort into exposed and unexposed groups for individual medication ingredients. Women with medication orders that overlapped with at least 1 day of pregnancy were considered exposed. While medication records may not accurately capture actual medication exposure, there is generally strong agreement between the medication use reported by pregnant women and their medication records [18]. We excluded medications that did not reach a minimum sample size of the exposed, which was 600. This minimum sample size was calculated using Epitools [19], with the following parameters: PTB prevalence rate of the PSJH maternity cohort (7.7%), assumed relative risk (1.55), desired level of confidence (0.9), and desired power for the detection of significant difference (0.8). The calculated minimum sample size was 582, but we rounded it to 600.

#### Outcomes

The primary outcome of interest was PTB, defined as gestational age at birth (GA; GA < 37 weeks). Secondary outcomes were low birth weight (LBW; birth weight < 2500 g) and small for gestational age (SGA; birth weight < 10th percentile of based on gestational age).

#### Covariates

We extracted maternal, pre-pregnancy, and prenatal characteristics and comorbidities information from EHR data. Pregnancy and maternal characteristics were collected during prenatal care or at time of delivery. These included parity, preterm history, delivery year, fetal sex, age at LMP, race, ethnicity, insurance status, pregravid body mass index (BMI), smoking, and use of alcohol and illegal drugs (Additional File 1: Table S1).

We conducted a parallel analysis with three different sets of covariates. First, we conducted propensity score matching with the covariates without comorbidities. Second, we addressed pre-pregnancy comorbidities based on the obstetric comorbidity index [20]. Selected comorbidities were renal diseases, chronic lung diseases, diabetes, leukemia, pneumonia, sepsis, cardiovascular diseases, sickle cell diseases, anemia, cystic fibrosis, and asthma (Additional File 1: Table S2). A similar practice was done in an at-scale study conducted by Sentinel System, one of the US Food and Drug Administration (FDA) efforts in surveillance medical products [21]. We excluded comorbidities specific to the prenatal period, such as gestational diabetes; the obstetric comorbidity index is designed to assess the mortality risk at delivery. Third, we selected the 25 most common comorbidities before and during the pregnancy (Additional File 1: Method S1). We acknowledge prenatal comorbidities do not satisfy the covariate definition. However, this study aims to explore the usefulness of EHRs and generate hypotheses. To do so, we employed an exploratory approach beyond the conventional one.

### Analysis

#### Descriptive statistics

**Fig. 1:**
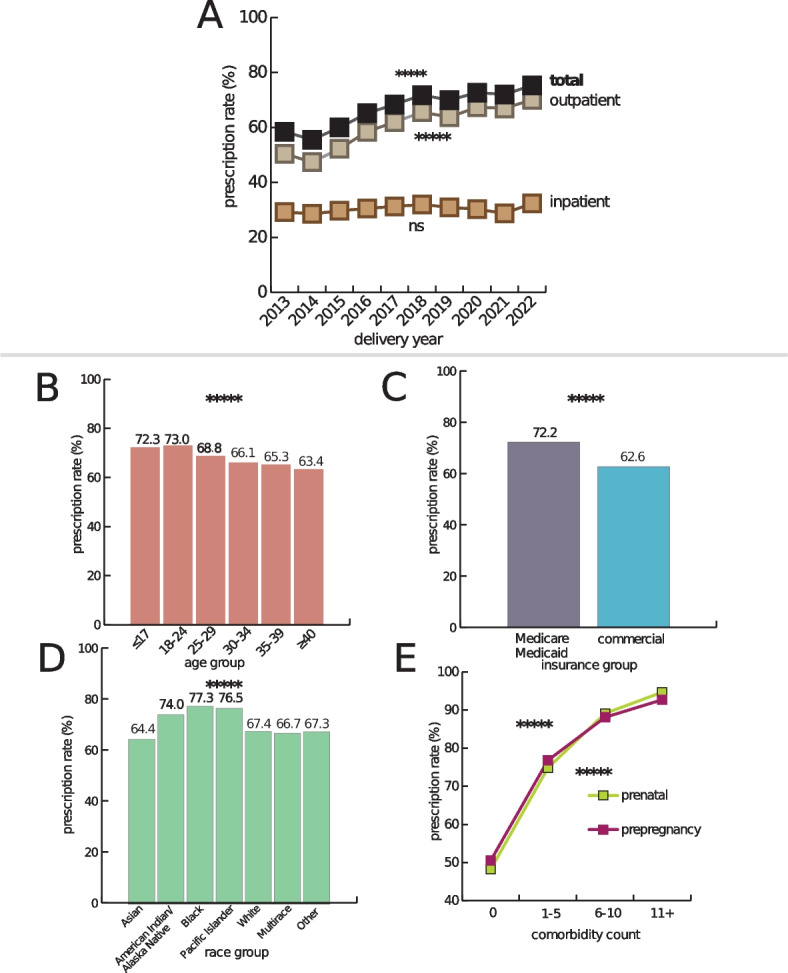
Overall prescription rate of PSJH pregnant population. **A** Plot shows the increase in total prescription rate from 2013 to 2022. The total medication prescription rate increased from 58.5 to 75.3% from 2013 to 2022 (*P* < 0.0001). The inpatient prescription rate increased from 29.3 to 32.4% (*P* = 0.2). In contrast, outpatient medication prescriptions increased from 50.5 to 70.1% (*P* < 0.0001). We evaluated the increase in prescription rate using linear regression. **B** Plot shows the total prescription rate across age groups (*P* < 0.0001). We evaluated the decrease in prescription rate across ages using linear regression. **C** Plot shows the difference in prescription rates between insurance groups (*P* < 0.0001). We evaluated the difference in prescription rate across categorical variables using the chi-square test. **D** Plot shows the difference in prescription rate across race groups (*P* < 0.0001). We evaluated the difference in prescription rate across categorical variables using the chi-square test. **E** Plot shows the increase in prescription rate based on comorbidity count. The increase in prescription rate across comorbidity count using linear regression

We described the source population on maternal characteristics, outcomes, and covariates. The descriptive statistics are presented in Additional File 1: Table S3. We characterized the prescription rate within the PSJH pregnant population in Fig. 1. We used the chi-square test and linear regression to evaluate the difference in prescription rate across categorical variables and continuous variables. Age distribution of this source population is described in Additional File 1: Fig. S2. Prescription patterns from 2013 to 2022 based on their ingredient and ATC classification categories are displayed in Additional File 1: Fig. S3.

#### Propensity score matching

We calculated the risk ratio of PTB, LBW, and SGA for individual outpatient medications that reached the minimum sample size. For each medication, the unexposed group was matched to the exposed group on the covariates. Missing values for parity and preterm history were imputed as 0. Missing values for pregravid BMI were imputed to be in the normal BMI category. The remaining covariates were imputed using the median. We used propensity score matching to account for covariates associated with adverse pregnancy outcomes. Compared to other propensity score methods and covariate adjustment methods, propensity score matching provided exceptional covariate balance across most circumstances [22]. An unsupervised learning model with *k*-nearest neighbors (*k* = 1), as recommended by a prior study [23], was used to match with replacement by the propensity logit metric. We evaluated the covariate balance using an average standardized mean difference. We excluded medication ingredients with an average standardized mean difference below 0.2. We categorized medications with statistically significant associations based on their indication in three categories: preterm labor (PTL) or PTB, PTB risk factors, and infection (Additional File 1: Table S4 [24–96]). Here, we considered association with a *P* value below 0.1 statistically significant. This is not a conventional practice in hypothesis-testing studies, but our study is hypothesis-generating. We are suggesting potential hypotheses for researchers to investigate further.

### Validation

We selected sertraline, acyclovir, and ferrous sulfate for further investigation. They had relatively large exposure groups and were statistically significant in an analysis adjusted for pre-pregnancy/prenatal common diagnoses. Details of the method are described in the Additional File 1: Method S2, Method S3, and Method S4.

#### Sertraline

Sertraline is a selective serotonin reuptake inhibitor (SSRI) antidepressant. Depression is a treatable disease and a risk factor for PTB [97]. We limited our analytic population to patients who had any depression diagnosis before pregnancy (Additional File 1: Table S2). We evaluated the risk of PTB in patients exposed to sertraline among patients who had depression onset before the pregnancy. Additionally, we assessed the likelihood of delivering preterm in patients exposed to SSRI within the same analytic population that we used to evaluate the risk of PTB in those exposed to sertraline.

#### Acyclovir

Acyclovir is a treatment for herpes virus infection, including shingles, chicken pox, and genital herpes. Genital herpes is a sexually transmitted disease, which is a risk factor for PTB. We determined the indication of treatment based on dosage [98]. According to the CDC treatment guideline [99], acyclovir is recommended starting at GA 36 weeks to suppress the reactivation of genital herpes among pregnant women. Patients who adhered to this treatment guideline delivered after 36 weeks of gestation, potentially introducing selection bias and leading to a lowered risk of PTB. Initially, we characterized the number of patients who initiated their prescription at 36 weeks of gestation to assess the proportion of patients following this CDC treatment guideline. Subsequently, we examined the likelihood of PTB in patients exposed to acyclovir before 36 weeks of gestation. We replicated the analysis on a subsample of patients who had indications of genital herpes (Additional File 1: Table S2). We then evaluated the risk of PTB among patients exposed to acyclovir or valacyclovir (oral prodrug of acyclovir) before 36 weeks of gestation.

#### Ferrous sulfate

Ferrous sulfate is a treatment for iron deficiency anemia, which is a risk factor for PTB. We assessed the impact of ferrous sulfate in the anemic group. The anemic group was determined based on the presence of iron-deficiency anemia diagnosis within 180 days before LMP to LMP (Additional File 1: Table S2).

## Results

### Descriptive statistics

We identified 365,075 patients as our analytic population who had continuously enrolled singleton pregnant patients. This population was enriched with people who were aged 30–34 (32.7%), White or Caucasian race (63.2%), non-Hispanic or Latino ethnicity (77.2%), Medicaid/Medicare insured, living in metropolitan areas (84.2%), and delivered in 2022 (12.4%). Median maternal age increases from 30.3 to 31.5 (*P* < 0.0001) from 2013 to 2022. The proportion of women aged 35 or older increased from 20.8 to 27.0% from 2013 to 2022 (Additional File 1: Fig. S2). The mean gestational age at delivery was 275.0 days. The average prevalence rates of PTB, SGA, and LBW were 7.7%, 12.1%, and 5.4% (Additional File 1: Table S3).

The total medication prescription rate increased from 58.5 to 75.3% from 2013 to 2022 (*P* < 0.0001). The inpatient prescription rate slightly increased from 29.3 to 32.4% (*P* = 0.2) In contrast, outpatient medication prescriptions increased from 50.5 to 70.1% (*P* < 0.0001) (Fig. 1). The maternal age group of 18–24 had the highest prescription rate of 73.0%. Mothers aged 40 or older had the lowest prescription rate reporting 63.4% (*P* < 0.0001). The Medicare/Medicaid insurance group had a higher prescription rate reporting 72.2%, than the commercial insurance group (62.6%; *P* < 0.0001). Amongst the race group, pregnant women who reported Black or African American race had the highest prescription rate of 77.3%, and Asian had the lowest, reporting 64.4% (*P* < 0.0001). We observed prescription rate increases as the number of comorbidities increased. This trend was similar for both pre-pregnancy and prenatal comorbidities. Approximately half of the pregnant people with no pre-pregnancy/prenatal problem diagnosis had a prescription during pregnancy. Patients with eleven or more pre-pregnancy/prenatal problem diagnoses had a prescription rate higher than 90% (Fig. 1).

### Propensity score matching

**Fig. 2:**
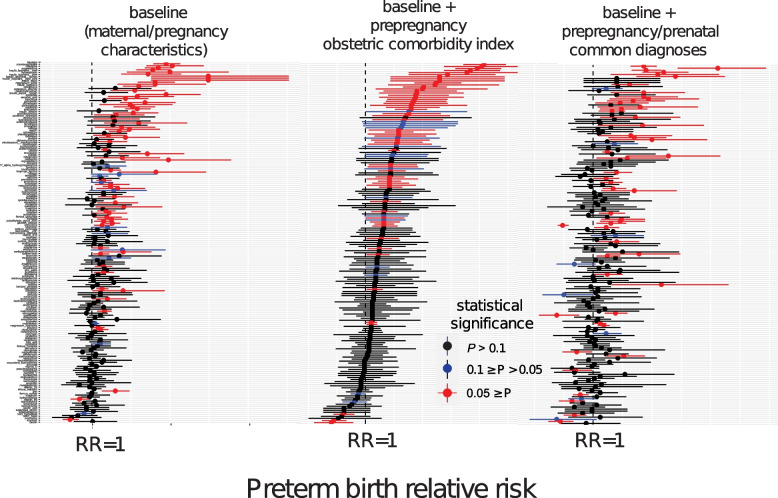
Forest plots of association between medication and risk of PTB. Left plot shows the forest plot of baseline analysis that adjusted maternal and pregnancy characteristics. The center plot shows a forest plot of analysis that adjusted for maternal and pregnancy characteristics and pre-pregnancy comorbidities from the obstetric comorbidity index. The right plot is a forest plot of analysis that adjusted for maternal and pregnancy characteristics and prenatal/pre-pregnancy common comorbidities. The *Y*-axis is the list of medications that met the minimum sample size in descending order of RR of analysis in the center plot. This figure is summarized in Table 1. RR, confidence interval, and *p*-values are reported in Additional File 2. Supplementary Data

**Fig. 3:**
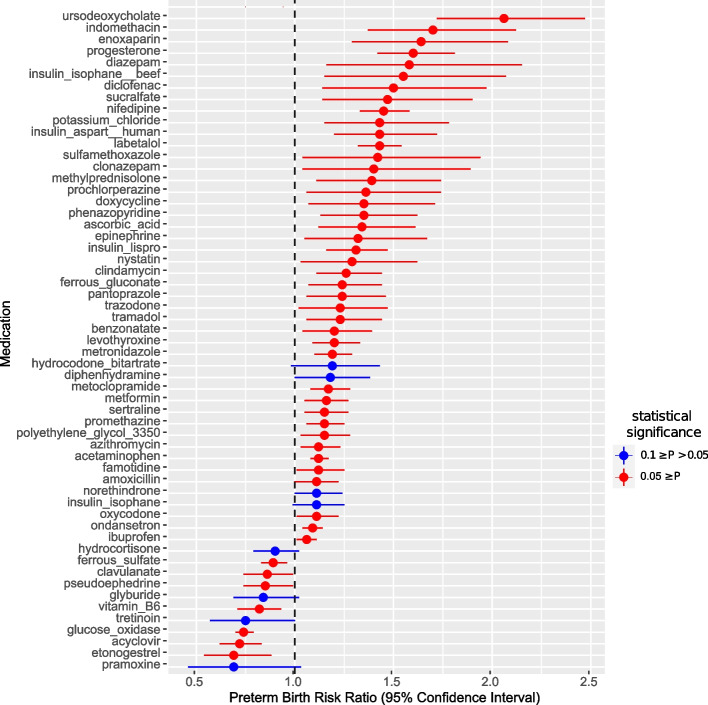
Forest plot of statistically significant association with risk of PTB. This plot is a forest plot of analysis that adjusted for maternal and pregnancy characteristics and prenatal/pre-pregnancy common comorbidities. Selection of prenatal and pre-pregnancy common comorbidities is described in Additional File 1: Method S1. RR, confidence interval, and p-values are reported in Additional File 2. Supplementary Data

From the initial pool of 1329 medications, 175 prenatally prescribed medications met the minimum sample size. None of the medications had an effect size below 0.2 after matching all three analyses. When we adjusted for baseline characteristics, pregnancy, and maternal characteristics, we identified a total of 76 (RR < 1: 20, RR ≥ 1:56) associations with a *p*-value below 0.1. The number of associations with statistical significance narrowed when additionally accounting for pre-pregnancy comorbidities in the obstetric comorbidity index. We observed 75 (RR < 1: 5, RR ≥ 1:70) medications associated with the risk of PTB with statistical significance. Finally, we identified 58 (RR < 1: 12, RR ≥ 1:46) medications associated with the risk of PTB in an analysis adjusted for common diagnoses during the pre-pregnancy and prenatal period (Fig. 2, Fig. 3, and Table 1). Statistically significant correlations were categorized into three categories based on their indication: PTL/PTB, risk factor of PTB, and infection (Additional File 1: Table S4) [24–96]. Forty-three medications had indications categorized into at least one category. Four medications fell into the category of PTL/PTB indication. Thirty-two medications had indications that were risk factors for PTB. Nine medications were prescribed in case of infections, including bacterial, fungal, and viral.

**Table 1:** Summary of associations based on statistical significance and relative risk

		**Baseline (maternal/pregnancy characteristics)**	**Baseline + prepregnancy comorbidity index**	**Baseline + prepregnancy/prenatal common comorbidity**
**RR < 1**	**0.05 ≥ *P***	4	3	8
	**0.1 ≥ *P* > 0.05**	16	2	4
	***P* > 0.1**	23	26	36
**RR ≥ 1**	**0.05 ≥ *P***	49	55	42
	**0.1 ≥ *P* > 0.05**	7	15	4
	***P* > 0.1**	77	75	82

### Validation

#### Sertraline

There were 29,352 patients who had depression diagnosis before the pregnancy. Respectively, 3214 and 5910 patients were exposed to sertraline or any SSRI. They were 1.28 times [1.14, 1.45] and 1.16 times [1.05, 1.28] more likely to deliver preterm than patients without exposure.

#### Acyclovir

The majority of patients (58.8%; 4947 out of 8420) who had prenatal acyclovir exposure started their prescription at or after 36 weeks of gestation. Those exposed to acyclovir before 36 weeks of pregnancy had 1.77 times (1.77 [1.52, 2.07]) higher likelihood of delivering preterm compared to patients without prenatal acyclovir exposure. However, within the subsample of patients diagnosed with genital herpes, we did not observe an elevated risk of PTB (OR = 1.19 [0.94, 1.50]). Additionally, there was no observed association between exposure to acyclovir and elevated risk of PTB when comparing individuals exposed to acyclovir and valacyclovir before 36 weeks of gestation (OR = 0.86 [0.74, 1.00]).

#### Ferrous sulfate

There were 774 patients diagnosed with iron deficiency anemia within a 180-day pre-pregnancy period. We observed 294 patients with a prescription for ferrous sulfate during pregnancy. Our analysis revealed no association between the prescription of ferrous sulfate and the risk of PTB (OR = 0.85 [0.48, 1.50]).

## Discussion

To our knowledge, this was the first study to use propensity score matching at scale on EHR to generate and prioritize testable hypotheses on drug effects associated with the risk of PTB. We retrospectively assessed 365,075 people who were continuously enrolled in PSJH. The majority of women took prescribed medication during pregnancy. From an initial pool of 1762 medications, we narrowed it down to 172 medications for hypothesis evaluation. Three of these detected signals were selected based on their relatively large exposure groups and statistical significance in an analysis adjusted for pre-pregnancy/prenatal common diagnoses. We investigated the heightened likelihood of delivering preterm associated with sertraline exposure and decreased chance related to acyclovir and ferrous sulfate exposures. We confirmed the association with sertraline, while the associations with acyclovir and ferrous sulfate lacked statistical significance.

We employed propensity score matching at scale on EHR and produced hypotheses for 172 medications. Among them, 57 of 172 mediations had statistically significant associations with the risk of PTB. There were a few prior studies with similar aims. Maric et al. (2019) [100] assessed administrative claims data on 2,538,255 deliveries and identified 863 medications with statistically significant associations. Their number of signals, statistically significant association, far exceeds ours because their sample size was greater and did not eliminate medication that did not meet the minimum sample size. That study had only 5 medications with an odds ratio below 1, whereas we had 12. Another effort to establish a framework to detect drug effect signals in maternal–fetal medicine was conducted by the Sentinel working group. Sentinel initiative, led by the US Food and Drug Administration (FDA), has created novel methods to evaluate the safety of approved medical products, including medications, vaccines, and devices. They used propensity score matching tree-based scan statistics methods on Medicaid data to discover infant outcomes associated with prenatal cephalosporin exposure in the first trimester [21]. That study utilized a different approach as they focused on multiple outcomes and single exposures; our study assessed single outcomes and multiple exposures. Both prior studies utilized claims data, whereas we used EHR.

The majority of patients were prescribed medications during pregnancy. This finding corresponds to observations in earlier studies. According to Mitchell et al. (2011) [11], in the US, 48% of women were exposed to prescribed medication during pregnancy in 2008. A systemic review study conducted on peer-reviewed literature from 1989 to 2010 in developed countries reported that 27% to 93% of pregnant women used prescription drugs, depending on the country [101]. In our study, we observed an increase in prenatal prescription rate from 58.5% to 75.3% from 2013 to 2022. This rate is higher than the prescription rate reported in 2008. The discrepancy in the prescription rate for medication during pregnancy may be attributable to a gradual increase in usage. Mitchell et al. in 2011 described an incremental increase in the use of prescription medications by 60% from 1986 to 2008. We also observed a rise in the prescription rate from 2013 to 2022. As discussed in the introduction, the common use of medication during pregnancy underscores the necessity to promote pharmacology research in pregnant women and to leverage already generated real-world data to expand our understanding of the efficacy and safety of medications during pregnancy.

Surprisingly, the prescription rate decreased as the maternal age increased. We first assumed that the increased prescription rate over the study period was attributable to increasing maternal age based on observation from Mitchell et al. (2011) [11]. Indeed, the median maternal age increased, and the proportion of women aged 30 or older gradually increased over our study period. However, the prescription rate did not correlate with the maternal age, contrary to our speculation. Women in the oldest age group, 40 or older, had the lowest prescription rate, whereas women aged 24 or younger had the highest. The major difference between our study and Mitchell et al. (2011) [11] is the study period and population. Their observation was based on 5008 deliveries from 1997 to 2003 in the US. In contrast, our observation was relatively similar to that from a more recent study [102] on 2.3 million patients who delivered live births from 2000 to 2019. Their study reported that the most prevalent medication exposures (antibacterial agents, antiemetics, and contraceptives) during pregnancy had a prescription pattern across age groups similar to our study. The younger group, 24 or younger, had much higher prescription rates for these medications than those of the older group, 35 or older. This counterintuitive finding regarding the decreasing prescription rate with age might be partly explained by the overall increase in prescription rates over time. Historically, there may have been more reluctance to take medication, but this hesitancy may be diminishing in recent years. Yet, older mothers may still retain some of this reluctance.

We employed traditional pharmacoepidemiology methods to evaluate the detected drug effect signals for sertraline, acyclovir, and ferrous sulfate. Specifically, we focused on assessing the negative association between sertraline/SSRI and the risk of PTB among patients who had an onset of depression before the pregnancy. We further validated this in a separate study [103]. We confirmed the correlation between exposure to sertraline/SSRI and the risk of PTB, and this correlation remained strong and significant through extensive sensitivity analyses. However, our study faced limitations in properly evaluating ferrous sulfate association with a lower risk of PTB due to the small sample size. Only 774 patients received a diagnosis of iron deficiency anemia within the 180-day pre-pregnancy period. Despite the small sample size of our study, a recent study reported that patients exposed to iron supplementation (ferrous sulfate, ferrous gluconate, ferrous fumarate, and ferrous glycinate) experienced reduced odds of preeclampsia and/or PTB [104].

In contrast to sertraline and ferrous sulfate, the signal we observed for acyclovir was misleading. According to CDC treatment guidelines [99], acyclovir is recommended for administration at 36 weeks of gestation for patients with genital herpes. This practice likely introduced selection bias, as the exposure group included patients who surpassed 36 weeks of gestation. In fact, 60% of patients were exposed to acyclovir at or after GA 36 weeks. When we restricted our exposure group to patients exposed to acyclovir before 36 weeks, the protective result associated with the risk of PTB disappeared. Interestingly, our result slightly differed from prior studies. In a previous study, exposure to valacyclovir, not to acyclovir, was associated with a lower risk of spontaneous PTB [100]. The investigation on sertraline and ferrous sulfate demonstrates the potential of our approach to produce and prioritize hypotheses to evaluate. However, misleading signals do exist. Thus, we must take a conservative stance and carefully verify detected drug effect signals.

We identified 118 medications with no statistical significance. Restricting our analyses to medications that satisfy minimum sample size ensures that associations lacking statistical significance are not dismissed as meaningless. Considering that pregnant women are typically excluded from clinical medication trials despite their medication use, the absence of an association with the risk of PTB is a valuable finding supporting the potential drug safety in relation PTB. It underscores the need for similar studies in pregnancy pharmacology to be conducted and repeated on real-world data to gather more evidence on medication’s safety, risks, and benefits for pregnant women.

We had one of the largest sample sizes for hypothesis-generating retrospective EHR studies for pregnant women. While similar studies exist, they often rely on claims data [21, 100]. Claims data may offer a larger sample size but EHR provides richer data on patient’s longitudinal health conditions, encompassing lab results, vital signs, and surveys [105]. Moreover, our study setting PSJH serves community hospitals/clinics in both rural and urban settings in seven western states in the US. This setting better reflects the general population better than the third-level academic hospital, which may focus more on high-risk pregnancies.

To ensure the integrity and reliability of our analyses, we implemented several measures to mitigate bias and ensure the robustness of our findings. We reduced the surveillance bias by restricting to continuously enrolled patients and leveraging propensity score matching. By limiting our study population to patients who were continuously enrolled, we excluded transient patients admitted for delivery who were likely to lack prenatal information. Furthermore, we mitigated the bias by matching patients in the treatment group to those in the control group with similar characteristics across covariates. Given that individuals exposed to medication may have more frequent doctor visits, ensuring comparability of patient health was crucial. Another noteworthy aspect of our approach was our commitment to evaluating all medications without introducing systemic bias. In research, there can be a tendency to focus on variables or hypotheses previously explored or considered more interesting. By conducting assessment on all medications that reached minimum sample size, we aimed to prevent such biases from influencing our analysis, which contributed to the overall rigor of our study.

One major limitation of this study was the absence of multiple testing corrections. We recognize that conducting multiple comparisons increases the likelihood of producing false positives. However, we deliberately did not correct for multiple testing, as the primary objective of this study was to produce hypotheses rather than to test them. Furthermore, different methods for multiple testing correction can yield varying adjusted *p*-values. Instead of applying specific correction methods, we presented confidence intervals. This decision allows future researchers to use them for meta-analysis, as recommended by a prior study [106]. We underscore the need for cautious consideration of these associations and advocate for thorough evaluation through meticulously designed studies that reflect the characteristics of exposures of interest and their indications.

Another limitation was the high number of missing values for pregravid BMI. To address this, we imputed pregravid BMI as normal, based on the assumption that the absence of this information suggested it was not a primary concern from the clinician’s perspective. However, we acknowledge that the distribution of pregravid BMI categories does not align with the national distribution for women of reproductive age. It is possible that some BMI information may be documented in unstructured notes, which could be extracted using Natural Language Processing (NLP) techniques. As this study is exploratory and aimed at generating hypotheses, we recommend that future research testing the generated hypothesis from this study address this limitation by conducting subgroup analysis focusing on patients with complete pregravid BMI information and leveraging NLP to collect more pregravid BMI information.

Another fundamental limitation of EHR is that medication records might not accurately reflect actual medication exposure. EHR do not capture over-the-counter medications unless the patient specifically reported them. This omission may lead to an underestimation of total medication use. Additionally, the EHR data do not provide information on whether prescribed medications were actually filled, which could result in an overestimation of medication adherence. Nevertheless, pregnant women are a unique clinical cohort with regular clinic visits and close monitoring by healthcare providers. Due to the potential risks to their fetus, pregnant women are especially vigilant about medication use [107] and generally demonstrate high agreement between self-reported medication use and recorded medication data [18]. While some bias may remain, we assume that women adhered to prescribed medications and that any discontinued medications were properly removed from the records. A further limitation is that route of administration was not considered in this analysis.

Lastly, the use of uniform sets of comorbidities is a limitation. Although we conducted multiple analyses with several groups of comorbidities, it is essential to note that individual medications are prescribed for specific indications. Medications with less common indications may not be adequately represented in the covariate we investigated. In the future, we can address this limitation by applying several promising approaches. One such approach is high-dimensional propensity score matching [108]. High-dimensional propensity score matching offers a robust way to control for confounding variables in observational studies. Unlike traditional propensity score matching, which considers a limited number of covariates, high-dimensional matching can involve hundreds of empirical covariates. Another promising approach is leveraging external databases such as ChEMBL. ChEMBL provides valuable information about drug indications, contraindications, and other clinical data. Leveraging external databases like ChEMBL enables researchers to automatically select relevant analytic cohorts and covariates relevant to drug indication and treatment.

## Conclusions

Most pregnant women are prescribed medication during pregnancy. This highlights the crucial need to advance pharmacology research in pregnant individuals, utilizing existing real-world data to enhance our knowledge of the safety of medications in pregnancy. We demonstrated the potential of using statistical data mining methods to generate and prioritize hypotheses on medication association with the risk of PTB. This foundational framework can be used for adverse outcomes such as gestational diabetes or preeclampsia. We note that these results should be further validated, reflecting the characteristics of exposures of interest and their indication. We only investigated drug effects associated with the risk of PTB. The mentioned drugs may be attributed to other adverse pregnancy outcomes or congenital disorders.

## Supplementary Information

Additional File 1. Supplementary Materials. Supplementary Methods S1-S4. Supplementary Tables S1-S4. Method S1- Selection of pre-pregnancy and prenatal common comorbidity. Method S2- Investigation on the association between sertraline/SSRI and elevated risk of PTB. Method S3-Investigation on the negative correlation between exposure to acyclovir and PTB risk. Method S4- Investigation on the association between exposure to ferrous sulfate and decreased risk of PTB. Table S1. Variable definition. Table S2. SNOMED diagnosis codes. Table S3. Descriptive statistics of source population. Table S4. Categorization of medications with statistically significant association with risk of PTB based on their indications.

Additional File 2: Supplementary Data. Results of large-scale propensity score matching analysis on preterm birth, low birth weight, and small for gestational age.

## Data Availability

All clinical logic has been shared in the manuscript and the GitHub repository (https://github.com/Hadlock-Lab/PSM_Maternity_at_scale). Results have been aggregated and reported within this paper to the extent possible while maintaining privacy from personal health information (PHI) as required by law. All data is archived within PSJH systems in a HIPAA-secure audited compute environment to facilitate verification of study conclusions.

## Abbreviations

ADR: Adverse drug reaction
CDC: Centers for Disease Control and Prevention
EHR: Electronic health record
FDA: Food and Drug Administration
GA: Gestational age
LBW: Low birth weight
LMP: Last menstrual period
OR: Odds ratio
PSJH: Providence St. Joseph Health
PTB: Preterm birth
PTL: Preterm labor
RR: Relative risk
SGA: Small for gestational age
SSRI: Selective serotonin reuptake inhibitor
US: United States

## References

[1] Lazarou J, Pomeranz BH, Corey PN. Incidence of adverse drug reactions in hospitalized patients: a meta-analysis of prospective studies. JAMA. 1998;279:1200–5. 9555760 10.1001/jama.279.15.1200

[2] Center for Drug Evaluation, Research. Preventable adverse drug reactions: a focus on drug interactions, https://www.fda.gov/drugs/drug-interactions-labeling/preventable-adverse-drug-reactions-focus-drug-interactions (accessed November 10, 2020).

[3] Hutchinson TA, Flegel KM, Kramer MS, et al. Frequency, severity and risk factors for adverse drug reactions in adult out-patients: a prospective study. J Chronic Dis. 1986;39:533–42. 3722316 10.1016/0021-9681(86)90198-0

[4] Hanlon JT, Schmader KE, Koronkowski MJ, et al. Adverse drug events in high risk older outpatients. J Am Geriatr Soc. 1997;45:945–8. 9256846 10.1111/j.1532-5415.1997.tb02964.x

[5] Gurwitz JH, Field TS, Harrold LR, et al. Incidence and preventability of adverse drug events among older persons in the ambulatory setting. JAMA. 2003;289:1107–16. 12622580 10.1001/jama.289.9.1107

[6] Overhage JM, Gandhi TK, Hope C, et al. Ambulatory computerized prescribing and preventable adverse drug events. J Patient Saf. 2016;12:69–74. 26001546 10.1097/PTS.0000000000000194

[7] Gandhi TK, Weingart SN, Borus J, et al. Adverse drug events in ambulatory care. N Engl J Med. 2003;348:1556–64. 12700376 10.1056/NEJMsa020703

[8] Feng C, Le D, McCoy AB. Using electronic health records to identify adverse drug events in ambulatory care: a systematic review. Appl Clin Inform. 2019;10:123–8. 30786301 10.1055/s-0039-1677738 PMC6382497

[9] Center for Drug Evaluation, Research. Clinical trials in pregnant women, https://www.fda.gov/drugs/development-resources/division-pediatric-and-maternal-health-clinical-trials-pregnant-women (accessed November 10, 2020).

[10] Fuhler GM. The immune system and microbiome in pregnancy. Best Pract Res Clin Gastroenterol. 2020;44–45:101671. 32359685 10.1016/j.bpg.2020.101671

[11] Mitchell AA, Gilboa SM, Werler MM, et al. Medication use during pregnancy, with particular focus on prescription drugs: 1976–2008. Am J Obstet Gynecol. 2011;205(51):e1-8. 10.1016/j.ajog.2011.02.029 PMC3793635 21514558

[12] Park MY, Yoon D, Lee K, et al. A novel algorithm for detection of adverse drug reaction signals using a hospital electronic medical record database. Pharmacoepidemiol Drug Saf. 2011;20:598–607. 21472818 10.1002/pds.2139

[13] Liu R, Zhang P. Towards early detection of adverse drug reactions: combining pre-clinical drug structures and post-market safety reports. BMC Med Inform Decis Mak. 2019;19:279. 31849321 10.1186/s12911-019-0999-1 PMC6918608

[14] Wu Y, Warner JL, Wang L, et al. Discovery of noncancer drug effects on survival in electronic health records of patients with cancer: a new paradigm for drug repurposing. JCO Clin Cancer Inform. 2019;3:1–9. 31141421 10.1200/CCI.19.00001 PMC6693869

[15] Liu S, Tang B, Chen Q, et al. Drug-drug interaction extraction via convolutional neural networks. Comput Math Methods Med. 2016;2016:6918381. 26941831 10.1155/2016/6918381 PMC4752975

[16] Rohani N, Eslahchi C. Drug-drug interaction predicting by neural network using integrated similarity. Sci Rep. 2019;9:13645. 31541145 10.1038/s41598-019-50121-3 PMC6754439

[17] Liu L, Johnson HL, Cousens S, et al. Global, regional, and national causes of child mortality: an updated systematic analysis for 2010 with time trends since 2000. Lancet. 2012;379:2151–61. 22579125 10.1016/S0140-6736(12)60560-1

[18] Howley MM, Fisher SC, Fuentes MA, et al. Agreement between maternal report and medical records on use of medications during early pregnancy in New York. Birth Defects Res. 2023;115:498–509. 36640121 10.1002/bdr2.2151 PMC12836282

[19] epitools. Sample size for a cohort study. Epitools, https://epitools.ausvet.com.au/cohortss (accessed September 14, 2023).

[20] Bateman BT, Mhyre JM, Hernandez-Diaz S, et al. Development of a comorbidity index for use in obstetric patients. Obstet Gynecol. 2013;122:957–65. 24104771 10.1097/AOG.0b013e3182a603bb PMC3829199

[21] Suarez EA, Nguyen M, Zhang D, et al. Sentinel Methods, https://www.sentinelinitiative.org/sites/default/files/Methods/Sentinel_Protocol_TreeScan_Pregnancy_V3.pdf (accessed August 9, 2023).

[22] Elze MC, Gregson J, Baber U, et al. Comparison of propensity score methods and covariate adjustment: evaluation in 4 cardiovascular studies. J Am Coll Cardiol. 2017;69:345–57. 28104076 10.1016/j.jacc.2016.10.060

[23] Austin PC. Statistical criteria for selecting the optimal number of untreated subjects matched to each treated subject when using many-to-one matching on the propensity score. Am J Epidemiol. 2010;172:1092–7. 20802241 10.1093/aje/kwq224 PMC2962254

[24] Luketic VA, Sanyal AJ. The current status of ursodeoxycholate in the treatment of chronic cholestatic liver disease. Gastroenterologist. 1994;2:74–9. 8055235

[25] Mawson AR. A role for the liver in parturition and preterm birth. J Transl Sci. 2016;2:154–9. 27595011 10.15761/JTS.1000133 PMC5010230

[26] Haas DM, Benjamin T, Sawyer R, et al. Short-term tocolytics for preterm delivery - current perspectives. Int J Womens Health. 2014;6:343–9. 24707187 10.2147/IJWH.S44048 PMC3971910

[27] Jupalli A, Iqbal AM. Enoxaparin. StatPearls Publishing, https://www.ncbi.nlm.nih.gov/books/NBK539865/ (2022, accessed September 13, 2023). 30969687

[28] Flickr F us on. What are the risk factors for preterm labor and birth? https://www.nichd.nih.gov/, https://www.nichd.nih.gov/health/topics/preterm/conditioninfo/who_risk (accessed September 13, 2023).

[29] Updated Clinical Guidance for the Use of Progesterone Supplementation for the Prevention of Recurrent Preterm Birth, https://www.acog.org/clinical/clinical-guidance/practice-advisory/articles/2023/04/updated-guidance-use-of-progesterone-supplementation-for-prevention-of-recurrent-preterm-birth (accessed September 13, 2023).

[30] Dhaliwal JS, Rosani A, Saadabadi A. Diazepam. StatPearls Publishing, https://www.ncbi.nlm.nih.gov/books/NBK537022/ (2022, accessed September 13, 2023). 30725707

[31] Männistö T, Mendola P, Kiely M, et al. Maternal psychiatric disorders and risk of preterm birth. Ann Epidemiol. 2016;26:14–20. 26586549 10.1016/j.annepidem.2015.09.009 PMC4688227

[32] Saleem F, Sharma A. NPH Insulin. StatPearls Publishing, https://www.ncbi.nlm.nih.gov/books/NBK549860/ (2023, accessed September 13, 2023). 31751050

[33] Crump C, Sundquist J, Sundquist K. Preterm birth and risk of type 1 and type 2 diabetes: a national cohort study. Diabetologia. 2020;63:508–18. 31802143 10.1007/s00125-019-05044-z PMC6997251

[34] Alfaro RA, Davis DD. Diclofenac. StatPearls Publishing, https://www.ncbi.nlm.nih.gov/books/NBK557879/ (2023, accessed September 13, 2023). 32491802

[35] Smith CJF, Förger F, Bandoli G, et al. Factors associated with preterm delivery among women with rheumatoid arthritis and women with juvenile idiopathic arthritis. Arthritis Care Res. 2019;71:1019–27. 10.1002/acr.23730 PMC6384155 30133181

[36] Van Calsteren K, Heyns L, De Smet F, et al. Cancer during pregnancy: an analysis of 215 patients emphasizing the obstetrical and the neonatal outcomes. J Clin Oncol. 2010;28:683–9. 19841323 10.1200/JCO.2009.23.2801

[37] Khan KM, Patel JB, Schaefer TJ. Nifedipine. StatPearls Publishing, https://www.ncbi.nlm.nih.gov/books/NBK537052/ (2023, accessed September 13, 2023).

[38] Conde-Agudelo A, Romero R, Kusanovic JP. Nifedipine in the management of preterm labor: a systematic review and metaanalysis. Am J Obstet Gynecol. 2011;204(134):e1-134.e20. 10.1016/j.ajog.2010.11.038 PMC3437772 21284967

[39] McMahon RS, Bashir K. Potassium Chloride. StatPearls Publishing, https://www.ncbi.nlm.nih.gov/books/NBK557785/ (2023, accessed September 13, 2023). 32491717

[40] Yang C-W, Li S, Dong Y. The prevalence and risk factors of hypokalemia in pregnancy-related hospitalizations: a nationwide population study. Int J Nephrol. 2021;2021:9922245. 34258064 10.1155/2021/9922245 PMC8261188

[41] Rubin R, Khanna NR, McIver LA. Aspart Insulin. StatPearls Publishing, https://www.ncbi.nlm.nih.gov/books/NBK500030/ (2022, accessed September 13, 2023). 29763206

[42] Kemnic TR, Coleman M. Trimethoprim Sulfamethoxazole. StatPearls Publishing, https://www.ncbi.nlm.nih.gov/books/NBK513232/ (2022, accessed September 13, 2023). 30020604

[43] Baer RJ, Nidey N, Bandoli G, et al. Risk of early birth among women with a urinary tract infection: a retrospective cohort study. AJP Rep. 2021;11:e5–14. 33489437 10.1055/s-0040-1721668 PMC7813589

[44] Basit H, Kahwaji CI. Clonazepam. StatPearls Publishing, https://www.ncbi.nlm.nih.gov/books/NBK556010/ (2023, accessed September 13, 2023). 32310470

[45] Rose MS, Pana G, Premji S. Prenatal maternal anxiety as a risk factor for preterm birth and the effects of heterogeneity on this relationship: a systematic review and meta-analysis. Biomed Res Int. 2016;2016:8312158. 27298829 10.1155/2016/8312158 PMC4889802

[46] Yonkers KA, Gilstad-Hayden K, Forray A, et al. Association of panic disorder, generalized anxiety disorder, and benzodiazepine treatment during pregnancy with risk of adverse birth outcomes. JAMA Psychiat. 2017;74:1145–52. 10.1001/jamapsychiatry.2017.2733 PMC5710298 28903165

[47] Ocejo A, Correa R. Methylprednisolone. StatPearls Publishing, https://www.ncbi.nlm.nih.gov/books/NBK544340/ (2022, accessed September 13, 2023). 31335060

[48] Crowley P. Prophylactic corticosteroids for preterm birth. Cochrane Database Syst Rev. 2000;2:CD000065. 10.1002/14651858.CD000065 10796110

[49] Din L, Preuss CV. Prochlorperazine. StatPearls Publishing, https://www.ncbi.nlm.nih.gov/books/NBK537083/ (2023, accessed September 13, 2023). 30725768

[50] Fabre C, Pauly V, Baumstarck K, et al. Pregnancy, delivery and neonatal complications in women with schizophrenia: a national population-based cohort study. Lancet Reg Health Eur. 2021;10:100209. 34806069 10.1016/j.lanepe.2021.100209 PMC8589714

[51] Patel RS, Parmar M. Doxycycline Hyclate. StatPearls Publishing, https://www.ncbi.nlm.nih.gov/books/NBK555888/ (2023, accessed September 13, 2023). 32310348

[52] Rours GIJG, Duijts L, Moll HA, et al. Chlamydia trachomatis infection during pregnancy associated with preterm delivery: a population-based prospective cohort study. Eur J Epidemiol. 2011;26:493–502. 21538042 10.1007/s10654-011-9586-1 PMC3115062

[53] Eastham JH, Patel P. Phenazopyridine. StatPearls Publishing, https://www.ncbi.nlm.nih.gov/books/NBK580545/ (2023, accessed September 13, 2023). 35593852

[54] Maxfield L, Crane JS. Vitamin C Deficiency. StatPearls Publishing, https://www.ncbi.nlm.nih.gov/books/NBK493187/ (2022, accessed September 13, 2023). 29630239

[55] Hauth JC, Clifton RG, Roberts JM, et al. Vitamin C and E supplementation to prevent spontaneous preterm birth: a randomized controlled trial. Obstet Gynecol. 2010;116:653–8. 20733448 10.1097/AOG.0b013e3181ed721d PMC2987650

[56] Dalal R, Grujic D. Epinephrine. StatPearls Publishing, https://www.ncbi.nlm.nih.gov/books/NBK482160/ (2023, accessed September 13, 2023). 29489283

[57] Kelly YJ, Brabin BJ, Milligan P, et al. Maternal asthma, premature birth, and the risk of respiratory morbidity in schoolchildren in Merseyside. Thorax. 1995;50:525–30. 7597666 10.1136/thx.50.5.525 PMC1021223

[58] Islam N, Khanna NR, Zito PM. Insulin Lispro. StatPearls Publishing, https://www.ncbi.nlm.nih.gov/books/NBK507840/ (2023, accessed September 13, 2023). 29939617

[59] Nystatin. National Institute of Diabetes and Digestive and Kidney Diseases, https://www.ncbi.nlm.nih.gov/books/NBK548581/ (2020, accessed September 13, 2023).

[60] Maki Y, Fujisaki M, Sato Y, et al. Candida Chorioamnionitis Leads to Preterm Birth and Adverse Fetal-Neonatal Outcome. Infect Dis Obstet Gynecol. 2017;2017:9060138. 29180840 10.1155/2017/9060138 PMC5664319

[61] Murphy PB, Bistas KG, Le JK. Clindamycin. StatPearls Publishing, https://www.ncbi.nlm.nih.gov/books/NBK519574/ (2023, accessed September 13, 2023). 30137858

[62] McDonald HM, O’Loughlin JA, Jolley P, et al. Vaginal infection and preterm labour. Br J Obstet Gynaecol. 1991;98:427–35. 2059587 10.1111/j.1471-0528.1991.tb10335.x

[63] Ferrous gluconate. Drugs.com, https://www.drugs.com/mtm/ferrous-gluconate.html (accessed September 13, 2023).

[64] Rahmati S, Azami M, Badfar G, et al. The relationship between maternal anemia during pregnancy with preterm birth: a systematic review and meta-analysis. J Matern Fetal Neonatal Med. 2020;33:2679–89. 30522368 10.1080/14767058.2018.1555811

[65] Bernshteyn MA, Masood U. Pantoprazole. StatPearls Publishing, https://www.ncbi.nlm.nih.gov/books/NBK499945/ (2023, accessed September 13, 2023).

[66] Lee K-S, Kim ES, Kim D-Y, et al. Association of Gastroesophageal Reflux Disease with Preterm Birth: Machine Learning Analysis. J Korean Med Sci. 2021;36:e282. 34751010 10.3346/jkms.2021.36.e282 PMC8575763

[67] Shin JJ, Saadabadi A. Trazodone. StatPearls Publishing, https://www.ncbi.nlm.nih.gov/books/NBK470560/ (2022, accessed September 13, 2023).

[68] Dhesi M, Maldonado KA, Maani CV. Tramadol. StatPearls Publishing, https://www.ncbi.nlm.nih.gov/books/NBK537060/ (2023, accessed September 13, 2023). 30725745

[69] Philip Thornton D. Benzonatate. Drugs.com, https://www.drugs.com/benzonatate.html (accessed September 13, 2023).

[70] Eghtedari B, Correa R. Levothyroxine. StatPearls Publishing, https://www.ncbi.nlm.nih.gov/books/NBK539808/ (2022, accessed September 13, 2023). 30969630

[71] Parizad Nasirkandy M, Badfar G, Shohani M, et al. The relation of maternal hypothyroidism and hypothyroxinemia during pregnancy on preterm birth: An updated systematic review and meta-analysis. Int J Reprod Biomed. 2017;15:543–52. 29662962 PMC5894470

[72] Isola S, Hussain A, Dua A, et al. Metoclopramide. StatPearls Publishing, https://www.ncbi.nlm.nih.gov/books/NBK519517/ (2023, accessed September 13, 2023). 30137802

[73] Nasri H, Rafieian-Kopaei M. Metformin: Current knowledge. J Res Med Sci. 2014;19:658–64. 25364368 PMC4214027

[74] Singh HK, Saadabadi A. Sertraline. StatPearls Publishing, https://www.ncbi.nlm.nih.gov/books/NBK547689/ (2023, accessed September 13, 2023). 31613469

[75] Southard BT, Al Khalili Y. Promethazine. StatPearls Publishing, https://www.ncbi.nlm.nih.gov/books/NBK544361/ (2022, accessed September 13, 2023). 31335081

[76] Dabaja A, Dabaja A, Abbas M. Polyethylene Glycol. StatPearls Publishing, https://www.ncbi.nlm.nih.gov/books/NBK557652/ (2023, accessed September 13, 2023). 32491584

[77] Sandman Z, Iqbal OA. Azithromycin. StatPearls Publishing, https://www.ncbi.nlm.nih.gov/books/NBK557766/ (2023, accessed September 13, 2023).

[78] Lim WS, Macfarlane JT, Colthorpe CL. Pneumonia and pregnancy. Thorax. 2001;56:398–405. 11312410 10.1136/thorax.56.5.398 PMC1746055

[79] Nguyen K, Dersnah GD, Ahlawat R. Famotidine. StatPearls Publishing, https://www.ncbi.nlm.nih.gov/books/NBK534778/ (2022, accessed September 13, 2023). 30521199

[80] Akhavan BJ, Khanna NR, Vijhani P. Amoxicillin. StatPearls Publishing, https://www.ncbi.nlm.nih.gov/books/NBK482250/ (2022, accessed September 13, 2023). 29489203

[81] Norethindrone. Drugs.com, https://www.drugs.com/mtm/norethindrone.html (accessed September 13, 2023).

[82] Grigoriadis S, Graves L, Peer M, et al. Maternal anxiety during pregnancy and the association with adverse perinatal outcomes: Systematic review and meta-analysis. J Clin Psychiatry. 2018;79:813. 10.4088/JCP.17r12011 30192449

[83] Sadiq NM, Dice TJ, Mead T. Oxycodone. StatPearls Publishing, https://www.ncbi.nlm.nih.gov/books/NBK482226/ (2022, accessed September 13, 2023). 29489158

[84] Griddine A, Bush JS. Ondansetron. StatPearls Publishing, https://www.ncbi.nlm.nih.gov/books/NBK499839/ (2023, accessed September 13, 2023). 29763014

[85] Ngo VTH, Bajaj T. Ibuprofen. StatPearls Publishing, https://www.ncbi.nlm.nih.gov/books/NBK542299/ (2023, accessed September 13, 2023).

[86] Hydrocortisone. Drugs.com, https://www.drugs.com/mtm/hydrocortisone.html (accessed September 13, 2023).

[87] Sinha S. Ferrous Sulfate: Uses, Dosage & Side Effects. Drugs.com, https://www.drugs.com/ferrous_sulfate.html (accessed September 13, 2023).

[88] Uto LR, Gerriets V. Clavulanic Acid. StatPearls Publishing, https://www.ncbi.nlm.nih.gov/books/NBK545273/ (2023, accessed September 13, 2023). 31424857

[89] Pseudoephedrine: medicine for a stuffy or blocked nose. nhs.uk, https://www.nhs.uk/medicines/pseudoephedrine/ (accessed September 13, 2023).

[90] Hardin MD, Jacobs TF. Glyburide. StatPearls Publishing, https://www.ncbi.nlm.nih.gov/books/NBK545313/ (2023, accessed September 13, 2023).

[91] Vitamin B6, https://ods.od.nih.gov/factsheets/VitaminB6-HealthProfessional/ (accessed September 13, 2023).

[92] Schmidt N, Gans EH. Tretinoin: A Review of Its Anti-inflammatory Properties in the Treatment of Acne. J Clin Aesthet Dermatol. 2011;4:22–9. 22125655 PMC3225141

[93] Liu B, Dai Q, Liu P, et al. Nanostructure-mediated glucose oxidase biofunctionalization for monitoring gestational diabetes. Process Biochem. 2021;110:19–25.

[94] Li D-K, Raebel MA, Cheetham TC, et al. Genital herpes and its treatment in relation to preterm delivery. Am J Epidemiol. 2014;180:1109–17. 25392064 10.1093/aje/kwu242

[95] Gilbert AL, Hoffman BL. Contraceptive Technology: Present and Future. Obstet Gynecol Clin North Am. 2021;48:723–35. 34756292 10.1016/j.ogc.2021.07.001

[96] Pramoxine. Memorial Sloan Kettering Cancer Center, https://www.mskcc.org/cancer-care/patient-education/medications/adult/pramoxine (accessed September 13, 2023).

[97] Grote NK, Bridge JA, Gavin AR, et al. A meta-analysis of depression during pregnancy and the risk of preterm birth, low birth weight, and intrauterine growth restriction. Arch Gen Psychiatry. 2010;67:1012–24. 20921117 10.1001/archgenpsychiatry.2010.111 PMC3025772

[98] Taylor M, Gerriets V. Acyclovir. StatPearls Publishing, https://www.ncbi.nlm.nih.gov/books/NBK542180/ (2023, accessed September 13, 2023). 31194337

[99] Herpes - Genital (HSV) / In Pregnancy, https://www.cdc.gov/stiapp/herpes-genital_pregnancy.html (2021, accessed September 15, 2023).

[100] Marić I, Winn VD, Borisenko E, et al. Data-driven queries between medications and spontaneous preterm birth among 2.5 million pregnancies. Birth Defects Res. 2019;111:1145–53. 31433567 10.1002/bdr2.1580 PMC11199711

[101] Daw JR, Hanley GE, Greyson DL, et al. Prescription drug use during pregnancy in developed countries: a systematic review. Pharmacoepidemiol Drug Saf. 2011;20:895–902. 21774029 10.1002/pds.2184 PMC3423446

[102] Suarez EA, Haug N, Hansbury A, et al. Prescription medication use and baseline health status of women with live-birth deliveries in a national data network. Am J Obstet Gynecol MFM. 2022;4:100512. 34656737 10.1016/j.ajogmf.2021.100512

[103] Hwang YM, Roper RT, Piekos SN, et al. Timing of selective serotonin reuptake inhibitor use and risk for preterm birth and related adverse events: with a consideration of the COVID-19 pandemic period. J Matern Fetal Neonatal Med. 2024;37:2313364. 38342572 10.1080/14767058.2024.2313364 PMC11033706

[104] Detlefs SE, Jochum MD, Salmanian B, et al. The impact of response to iron therapy on maternal and neonatal outcomes among pregnant women with anemia. Am J Obstet Gynecol MFM. 2022;4:100569. 35033748 10.1016/j.ajogmf.2022.100569 PMC10446247

[105] Huybrechts KF, Bateman BT, Hernández-Díaz S. Use of real-world evidence from healthcare utilization data to evaluate drug safety during pregnancy. Pharmacoepidemiol Drug Saf. 2019;28:906–22. 31074570 10.1002/pds.4789 PMC6823105

[106] Rothman KJ. No adjustments are needed for multiple comparisons. Epidemiology. 1990;1:43–6. 2081237

[107] Obi OC, Anosike C. A cross-sectional study on the knowledge, attitude, and practice of pregnant women regarding medication use and restriction during pregnancy. Explor Res Clin Soc Pharm. 2023;11:100308. 37533757 10.1016/j.rcsop.2023.100308 PMC10392609

[108] Schneeweiss S, Rassen JA, Glynn RJ, et al. High-dimensional propensity score adjustment in studies of treatment effects using health care claims data. Epidemiology. 2009;20:512–22. 19487948 10.1097/EDE.0b013e3181a663cc PMC3077219

