# Supplementary material for: Accelerating adverse pregnancy outcomes research amidst rising medication use: parallel retrospective cohort analyses for signal prioritization
Source: BMC Med. 2024 Oct 25;22:495. doi: 10.1186/s12916-024-03717-0 (PMC11520034; doi:10.1186/s12916-024-03717-0)
Supplement: Supplementary file 1 — Additional File 1. Supplementary Materials. Supplementary Methods S1-S4. Supplementary Tables S1-S4. Method S1- Selection of pre-pregnancy and prenatal common comorbidity. Method S2- Investigation on the association between sertraline/SSRI and elevated risk of PTB. Method S3-Investigation on the negative correlation between exposure to acyclovir and PTB risk. Method S4- Investigation on the association between exposure to ferrous sulfate and decreased risk of PTB. Table S1. Variable definition. Table S2. SNOMED diagnosis codes. Table S3. Descriptive statistics of source population. Table S4. Categorization of medications with statistically significant association with risk of PTB based on their indications. [file 12916_2024_3717_MOESM1_ESM.docx]

**SUPPLEMENTARY METHODS**

**Method S1** Selection of pre-pregnancy and prenatal common diagnosis

We collected data on the 25 most common diagnoses before pregnancy and during pregnancy based on the SNOMED code. Diagnoses that share similar clinical contexts or medication treatment. For example, Type I and Type II diabetes were grouped together as diabetes. Another example includes grouping anxiety and depression together. The prepregnancy diagnoses included in the analysis were diabetes, anemia, bacterial infection, vitamin D deficiency, gastroesophageal reflux diseases, irregular periods, fatigue, asthma, anxiety/depression, hypertensive disorder, hypothyroidism, and insomnia. The prenatal diagnoses included were history of caesarean section, diabetes, preterm labor, anemia, bacterial infection, RhD negative, breech presentation, abnormal fetal movement, hypertensive disorder, poor fetal growth, placental previa, anxiety/depression, excessive fetal growth, premature rupture of membranes. SNOMED codes are listed in Additional File 1: Table S2.

**Method S2** Investigation on the association between exposure to sertraline/SSRI and elevated risk of PTB

We limited our analytic population to patients with at least one depression diagnosis before the pregnancy (Additional File 1: Table S2). We calculated the risk of PTB using the propensity score adjustment method. We chose propensity score adjustment because it performs well compared to other covariate adjustment methods^22^, and our analytic population shares similar characteristics and is balanced. Propensity score was calculated on maternal and pregnancy characteristics. This includes delivery year, parity, gravidity, maternal age, race, ethnicity, preterm history, ethanol consumption status, smoking status, and illegal drug use status.

**Method S3** Investigation on negative correlation between exposure to acyclovir and PTB risk

We first assessed the risk of PTB in patients exposed to acyclovir before the GA of 36 weeks using propensity score matching. We used matching here because the control and treatment groups were highly imbalanced. Second, we calculated the chance of delivering before 37 weeks on a subsample of patients with an indication of genital herpes, determined based on a diagnosis before delivery (Table S2). Third, we compared PTB risk between patients exposed to acyclovir and valacyclovir before GA 36 weeks. We used the propensity score adjustment method for the second and third analyses because the control and treatment groups were balanced; both the control and treatment groups had genital herpes. Propensity score was calculated on maternal and pregnancy characteristics. This includes delivery year, parity, gravidity, maternal age, race, ethnicity, preterm history, ethanol consumption status, smoking status, and illegal drug use status.

**Method S4** Investigation on the association between exposure to ferrous sulfate and decreased risk of PTB

We limited our analytic population to patients diagnosed with iron deficiency anemia (Additional File 1: Table S2) within 180 days of the prepregnancy period.  We used propensity score adjustment because the control and treatment groups were balanced; both had iron deficiency anemia before pregnancy. The propensity score was calculated on maternal/pregnancy characteristics. This includes delivery year, parity, gravidity, maternal age, race, ethnicity, preterm history, ethanol consumption status, smoking status, and illegal drug use status.

**SUPPLEMENTARY TABLES**

**Table S1** Variable definition

| **Category** | **Variable** | **Definitions** |
| --- | --- | --- |
| **Maternal/pregnancy characteristics** | Race | Race noted in medical record. Missing values were encoded as Unknown; American Indian or Alaska Native, Asian, Black or African American, Native Hawaiian or Other Pacific Islander, White or Caucasian, Multiracial, Other, Unknown |
|  | Ethnicity | Ethnicity noted in medical record. Missing values were encoded as unknown; Hispanic or Latino, Not Hispanic or Latino, Unknown |
|  | Maternal age | Maternal age at the start of pregnancy; 18 ~ 24, 25 ~ 29, 30 ~ 34, 35 ~ 40, 41 ~ 44 |
|  | Pregravid BMI | Pregravid Body Mass Index (kg/m2). Missing value was encoded as unknown; Underweight (<18.5 BMI), Normal (18.5 - 24.9 BMI), Overweight ( 25.0 - 29.9 BMI), Obese (>30.0) |
|  | Insurance status | Commercial, Medicaid, Medicare, Uninsured-Self-Pay |
|  | Smoker | Self-reported smoking status; 0,1 |
|  | Illegal drug use | Self-reported illegal drug use status; 0,1 |
|  | Preterm history | History of preterm delivery; 0,1 |
|  | Parity | Number of times a patient has delivered a fetus older than 20 weeks of gestation prior to the current pregnancy; 0, 1~5, 6 |
|  | Gravidity | Number of times a patient has been pregnant; 0, 1~5, 6 |
|  | Delivery year | Year of delivery; 2013~2022 |
| **Maternal-fetal health outcomes** | Low birth weight | Infant birth weight ≤ 2,500g |
|  | Preterm birth | Infant gestational age (GA) at birth < 37 weeks |
|  | Small for gestational age | Infant birth weight < 10th percentiles for infants of same GA |

BMI Body Mass Index; CDC Center for Disease Control and Prevention; GA Gestational age; SVI Social Vulnerability Index

**Table S2** SNOMED diagnosis codes

| **Diagnosis** | **SNOMED Concept ID** |
| --- | --- |
| Abnormal fetal movement | 276369006, 363093002, 364755008 |
| Anemia | 271737000 |
| Anxiety | 48694002 |
| Asthma | 195967001 |
| Bacterial infection | 87628006,442635007,170488007, 710954001 |
| Breech presentation | 6096002 |
| Cardiovascular diseases | 49601007 |
| Chronic kidney diseases | 709044004 |
| Cystic fibrosis | 190905008 |
| Depression | 35489007 |
| Diabetes | 73211009 |
| Excessive fetal growth | 22173004, 199616008 |
| Fatigue | 84229001 |
| Gastroesophageal reflux diseases | 235595009 |
| History of cesarean section | 161805006 |
| Hypertensive disorder | 10725009,720568003,48146000,59621000,429198000, 71701000119105,71421000119105, 397748008, 706882009, 697929007, 1078301000112109, 206596003, 23130000, 5501000119106, 118781000119108, 84094009, 31992008, 38341003, 24184005 69909000, 198941007,  367390009, 48194001 |
| Intrauterine device birth control | 737288007 |
| Insomnia | 193462001 |
| Irregular period | 80182007, 237055002 (polycystic ovary syndrome) |
| Leukemia | 93143009 |
| Placenta previa | 36813001, 445122007 |
| Pneumonia | 233604007 |
| Poor fetal growth | 2033007, 397949005 |
| Preterm labor | 289733005, 6383007 |
| Premature rupture of membrane | 44223004 |
| Rhesus D negative | 165746003 |
| Sepsis | 91302008 |
| Sickle cell diseases | 417357006 |
| Vitamin D deficiency | 34713006 |

All descendant SNOMED codes are included

**Table S3** Descriptive statistics of source population

|  | **Source population (n=365,075)** |
| --- | --- |
| **Maternal-fetal health outcome** |  |
| **Gestational days** | 275.0 (12.8) |
| **Preterm birth** | 28185 (7.7) |
| **Small for gestational age** | 43905 (12.1) |
| **Low birth weight** | 19611 (5.4) |
| **Delivery method** |  |
| Vaginal | 252001 (69.0) |
| C-section | 110386 (30.2) |
| Other | 277 (0.1) |
| Missing | 2411 (0.7) |
| **Maternal/pregnancy characteristics** |  |
| **Maternal age** | 31.0 (5.8) |
| **Age group** |  |
| 17 or younger | 3112 (1.1) |
| 18-24 | 61214 (22.1) |
| 25-29 | 93220 (33.6) |
| 30-34 | 119530 (43.1) |
| **Race group** |  |
| American Indian or Alaska Native | 4764 (1.3) |
| Asian | 29448 (8.1) |
| Black or African American | 15478 (4.2) |
| Native Hawaiian or Other Pacific Islander | 4249 (1.2) |
| White or Caucasian | 230765 (63.2) |
| Multirace | 17932 (4.9) |
| Other | 60175 (16.5) |
| Missing | 2264 (0.6) |
| **Ethnic group** |  |
| Hispanic/Latino | 75271 (20.6) |
| Not Hispanic/Latino | 281675 (77.2) |
| Unknown/Not Reported | 8061 (2.2) |
| **BMI category** |  |
| Underweight | 3002 (0.8) |
| Normal | 52389 (14.4) |
| Obese | 31243 (8.6) |
| Overweight | 31300 (8.6) |
| Missing | 247141 (67.7) |
| **Insurance** |  |
| Commercial | 170164 (46.6) |
| Medicaid/Medicare | 192453 (52.7) |
| Self Pay | 84 (0.0) |
| Missing | 2374 (0.7) |
| **Smoker** | 26108 (7.2) |
| **Illegal drug user** | 27071 (7.4) |
| **Alcohol user** | 64577 (17.7) |
| **Vulnerability index of socioeconomic status** | 0.4 (0.2) |
| **Vulnerability index of housing composition** | 0.4 (0.3) |
| **Vulnerability index of minority status and language** | 0.6 (0.2) |
| **Vulnerability index of housing type and transportation** | 0.6 (0.3) |
| **Rural/urban classification** |  |
| Metropolitan | 307464 (84.2) |
| Micropolitan | 17425 (4.8) |
| Small Town | 5871 (1.6) |
| Rural | 4567 (1.3) |
| Missing | 29747 (8.1) |
| **Gravidity** |  |
| 1 | 82176 (22.5) |
| 2-4 | 198452 (54.4) |
| 5≤ | 42011 (11.5) |
| Missing | 42436 (11.6) |
| **Parity** |  |
| 0 | 45275 (12.4) |
| 1 | 127735 (35.0) |
| 2-4 | 141207 (38.7) |
| 5≤ | 8422 (2.3) |
| Missing | 42436 (11.6) |
| Preterm history |  |
| Yes | 76632 (21.0) |
| No | 246007 (67.4) |
| Missing | 42436 (11.6) |
| **Fetal sex** |  |
| Female | 164049 (48.6) |
| Male | 173160 (51.3) |
| Unknown | 51 (0.0) |
| Other | 16 (0.0) |
| **Delivery year** |  |
| 2013 | 20799 (5.9) |
| 2014 | 30834 (8.8) |
| 2015 | 34844 (10.0) |
| 2016 | 36288 (10.4) |
| 2017 | 35895 (10.3) |
| 2018 | 35587 (10.2) |
| 2019 | 36953 (10.6) |
| 2020 | 35431 (10.1) |
| 2021 | 39791 (11.4) |
| 2022 | 43188 (12.4) |

**Table S4** Categorization of medications with statistically significant association with risk of PTB based on their indication

| **Medication** | **Usage/usage during pregnancy** | **Indication** | | |
| --- | --- | --- | --- | --- |
|  |  | **Preterm labor/birth** | **PTB risk factor** | **Infection** |
| ursodeoxyholate | Dissolve gallstones. Treat cholestatic liver diseases^24^ | N | Y(liver disorder^25^) | N |
| indomethacin | Pain treatment. Tocolytics for preterm labor^26^ | Y^26^ | N | N |
| enoxaparin | Anticoagulation^27^ | N | Y(blood clot^28^) | N |
| progesterone | Treatment option for preterm birth^29^ | Y | N | N |
| diazepam | Treat anxiety, muscle spasm, and seizures^30^ | N | Y(anxiety^31^) | N |
| insulin isophane beef | Insulin given to help control blood sugar levels in people with diabetes^32^ | N | Y(diabetes^33^) | N |
| diclofenac | Treat pain, migraines, and arthritis^34^ | N | Y (arthritis^35^) | N |
| sucralfate | Medication used to treat duodenal ulcers, chemotherapy-induced mucostasis | N | Y(cancer^36^) | N |
| nifedipine | Treat high blood pressure^37^ | Y^38^ | Y (diabete^33^) | N |
| potassium chloride | Treat and prevent low blood potassium^39^ | N | Y(hypokalemia^40^) | N |
| insulin aspart human | Treat diabetes^41^ | N | Y(diabetes^33^) | N |
| sulfamethoxazole | Antibiotics used for bacterial infection such as urinary tract infection^42^ | N | Y(urinary tract infection^43^) | Y |
| clonazepam | Sedative. Treatment for seizures, panic disorder, and anxiety^44^ | N | Y(anxiety^45^, panic^46^) | N |
| methylprednisolone | Treatment for inflammation, flares of chronic illness^47^ | Y^48^ | N | N |
| prochlorperazine | Treatment for nausea, vomiting, anxiety, and schizophrenia^49^ | N | Y(schizophrenia^50^) | N |
| doxycycline | Antibiotics used for bacterial infection including acne, urinary tract infection, gonorrhea, chlamydia, etc^51^ | N | Y(chlamydia^52^) | Y |
| phenazopyridine | Relieve symptoms caused by urinary tract infection^53^ | N | Y(urinary tract infection^43^) | Y |
| ascorbic acid | Treat vitamin c deficiency^54^ | N | Y^55^ | N |
| epinephrine | Treatment for severe asthma attack and allergic reactions^56^ | N | Y(asthma^57^ | N |
| insulin lispro | Treament for diabetes^58^ | N | Y^33^ | N |
| nystatin | Treat fungal infection^59^ | N | Y(candida^60^) | Y |
| clindamycin | Antibiotic for skin and vaginal infection^61^ | N | Y^62^ | Y |
| ferrous gluconate | Used to treat or prevent iron deficiency anemia^63^ | N | Y(anemia^64^) | N |
| pantoprazole | Reduce the amount of stomach acid. Used for heartburn, acid reflux, gastroesophageal reflux diseases^65^ | N | Y(gerd^66^) | N |
| trazodone | Antidepresssant^67^ | N | Y(depression^97^) | N |
| tramadol | Strong pain medication^68^ | N | N | N |
| benzonatate | Treat cough caused by common cold and other breathing problems(asthma, pneumonia)^69^ | N | Y^57^ | N |
| levothyroxine | Treat hypothyroidism^70^ | N | Y(hypothyroidism^71^) | N |
| metoclopramide | Treat GERD, and gastroparesis in patients with diabetes^72^ | N | Y^66^ | N |
| metformin | Treat type 2 diabetes^73^ | N | Y(diabetes^33^) | N |
| sertraline | Antidepressant, selective serotonin reuptake inhibitor^74^ | N | Y(depression^31^) | N |
| promethazine | Antihistamine treating allergies and motion sickness^75^ | N | N | N |
| polyethylene glycol 3350 | Treat occasional constipation^76^ | N | N | N |
| azithromycin | Antibiotic medicine treating pneumonia, ear, nose, throat, and sinus^77^ | N | Y(pneumonia^78^) | Y |
| acetaminophen | Treat minor aches, pains, and fevers^109^ | N | N | N |
| famotidine | Used to treat gerd^79^ | N | Y(gerd^66^) | N |
| amoxicillin | Penicillin antibiotic treating infection and stomach ulcers^80^ | N | N | Y |
| norethindrone | Used to prevent pregnancy^81^ | N | N | N |
| insulin isophane | Control blood sugar with diabetes^32^ | N | Y(diabetes^33^) | N |
| oxycodone | Treat moderate to severe pain^83^ | N | N | N |
| ondansetron | Prevent nausea and vomiting^84^ | N | N | N |
| ibuprofen | Treat fever and mild to severe pain^85^ | N | N | N |
| hydrocortisone | Calm down body's immune response to reduce pain, itching, swelling, inflammation^86^ | N | N | N |
| ferrous sulfate | Iron supplement used for iron deficient anemia^87^ | N | Y^64^ | N |
| clavulanate | Used in conjunction with amoxicillin to treat certain bacterial infection^88^ | N | N | Y |
| pseudoephedrine | Treat stuffy nose and sinuses^89^ | N | N | N |
| glyburide | Treat type 2 diabetes^90^ | N | Y^33^ | N |
| vitamin b6 | Vitamin for healthy nervous system and immune system^91^ | N | N | N |
| tretinoin | Acne treatment^92^ | N | N | N |
| glucose oxidase | Probe for sensing gestational diabetes^93^ | N | N | N |
| acyclovir | Treat herpes virus infection^98^ | N | Y(genital herpes^94^) | Y |
| etonogestrel | Birth control^95^ | N | N | N |
| pramoxine | Relieve pain and itching from insect bites or hemorrhoids^96^ | N | N | N |
